# Context-specific advancements in service delivery for communication disorders in South Africa

**DOI:** 10.4102/sajcd.v70i1.1021

**Published:** 2023-12-20

**Authors:** Anita Edwards, Faheema Mahomed-Asmail, Anna-Mari Olivier, Jeannie van der Linde

**Affiliations:** 1 South African Speech Language and Hearing Association (SASLHA), Durban, South Africa; 2 Department of Speech-Language Pathology and Audiology, Faculty of Humanities, University of Pretoria, Pretoria, South Africa

The *South African Journal of Communication Disorders* (SAJCD) continues to contribute to the field of speech-language pathology, dysphagia and audiology within developing countries. This is achieved through peer-reviewed publications that explore innovative approaches aimed at addressing the diverse needs of population groups with communication disorders. In this edition, several articles shed light on critical aspects of clinical practice, accessibility to therapy, and improving client outcomes in the following areas: stuttering, dysphagia, reading interventions and accessibility to hearing healthcare services.

Two of the articles are in the stuttering domain. The first study focuses on the motivations of people who stutter (PWS) attending stuttering support groups, contributing by providing insight into the perspectives of PWS and thus informing clinical practice. The second study examines an innovative approach to stuttering therapy using eye movement. The results suggest that this approach can be easily translated into any language and provide a service delivery model that may be culturally and linguistically appropriate in diverse populations. Furthermore, the remote delivery capability of this approach makes stuttering therapy more available to a broader range of individuals.

Four articles in this edition delve into dysphagia and related aspects of swallowing. One article was written from the perspective of nurses and introduces the knowledge-to-action process model. This model makes use of knowledge translation in oral care in South Africa. Its contribution lies in demonstrating the value of collaboration between the oral care treating team members, which can lead to greater sustained use of intervention strategies.

A second article, a scoping review, related to dysphagia in the context of human immunodeficiency virus (HIV) and/or acquired immunodeficiency syndrome (AIDS), emphasises the importance of speech-language pathologists in monitoring swallowing during mealtimes and pill swallowing. This involvement can reduce the risk of nutritional compromise and non-compliance with medication. This article underscores the critical role of speech-language pathologists in advocating for comprehensive patient care and the importance of their integration into the care team. Concerning dysphagia patients’ access to care, the third article weighs up the pros and cons of dysphagia triage in assessing therapy needs. This study critically evaluates the dysphagia triage checklist development and implementation in South Africa. While the current checklist may not be recommended for use in its current form, the research provides a valuable platform for further refinement to optimise patient care. The article emphasises the necessity of considering contextual, economic, technical, and logistic aspects when implementing dysphagia triage. Finally, a fourth article relating to this topic challenges speech-language therapists who work in the field of dysphagia and among those affected by advanced dementia, to be aware of the ongoing need for clinical supervision, mentoring, and continuous professional development to improve knowledge and decision-making.

In the field of language development, the article on shared reading with caregivers demonstrates how a simple and cost-effective intervention can positively impact caregiver perceptions and their shared reading practices. Similarly, in the field of developmental delays in young children, an article on Enhanced Milieu Teaching reports on the feasibility and acceptability of adapting a clinical programme to support families in resource-limited settings.

Regarding hearing-related research, two topics are addressed. Firstly, regarding access to care, which in turn has the potential to influence hearing healthcare policies. Secondly, an article that improves our knowledge and awareness of patients’ attitudes and experiences, which in turn inform clinical practice.

Access to hearing healthcare for vulnerable populations is explored in the article on the experience with bone-conduction hearing aids in the Western Cape. This article outlines strategies employed at a children’s hospital to enhance accessibility to hearing devices. Immediate fitting of bone conduction hearing devices on a softband after a hearing loss diagnosis and remote follow-up via technology are recommended as innovative strategies for this patient population.

The second article on this topic describes the implementation of a Newborn Hearing Screening Programme in a public hospital. The authors reflect on lessons learnt and describe the processes in managing a large-scale project.

The third article on access to hearing healthcare reports that more than half of those eligible for follow-up hearing healthcare do not return for their appointments. An important influencing factor is reported to be the attitude of the staff in the hearing healthcare clinic. The findings inform clinical planning as well as policy formulation on hearing healthcare services in the public healthcare sector.

The second group of audiology-specific articles relate to audiologists’ understanding of patients’ hearing loss experience. One article highlights the attitudes and beliefs about Noise Induced Hearing Loss (NIHL). Different approaches to Hearing Conservation Programmes in coal mines are needed as participants have positive attitudes towards prevention of NIHL and the use of hearing protection devices and yet miners are still losing their hearing.

Another article reports that the inclusion of siblings in aural rehabilitation and family engagements facilitates more effective outcomes for the child with a hearing loss. Another article also emphasises the need for person-centred and family-centred care improving the quality of life after a diagnosis of sudden onset hearing loss. These approaches also facilitated improved outcomes of management strategies for the debilitating condition of sudden onset hearing loss.

The final category of articles in this edition addresses higher education and training in the field of Speech-Language Pathology and Audiology in South Africa. The studies not only sheds light on the challenges but also offer a path forward towards a more inclusive and representative Speech-Language and Audiology profession in South Africa, aligned with the principles of decolonisation and Afrocentrism.

These articles collectively demonstrate the dynamic nature of the topics addressed through this journal and its potential to positively impact the lives of individuals with communication disorders. From incorporating patient perspectives to innovative therapies and models of care, these contributions highlight the vital role of research informing clinical practice and increasing the accessibility of services to all clients with communication needs.

